# Walking with noise: the impact of auditory white noise stimulation on gait dynamics in young and older adults

**DOI:** 10.3389/fbioe.2026.1910987

**Published:** 2026-10-07

**Authors:** Michelle Gwerder, Yong K. Kim, Paciane B. Studer, Angela Frautschi, William R. Taylor, Navrag B. Singh

**Affiliations:** 1 Laboratory for Movement Biomechanics, Department of Health Sciences and Technology, ETH Zürich, Zürich, Switzerland; 2 Future Health Technologies, Singapore-ETH Centre, Campus for Research Excellence and Technological Enterprise (CREATE), Singapore, Singapore

**Keywords:** adaptation, auditory white noise stimulation, fall risk, stability, stochastic resonance

## Abstract

**Background:**

Walking is essential for maintaining autonomy and quality of life, yet age-related declines in sensory and motor function can impair gait control and increase fall risk. Auditory white noise stimulation may offer a simple approach to support sensorimotor function and enhance gait strategy. This effect may be particularly relevant in older adults, because their sensory signals are often diminished. This intervention study tested three hypotheses: 1) subthreshold auditory white noise stimulation enhances gait strategy more in older than in younger adults, 2), subthreshold auditory white noise stimulation has a greater impact on gait strategy than threshold and supra-threshold noise, and 3) the effects of auditory white noise persist beyond the period of stimulation.

**Methods:**

Fifty young (mean age 23.8 ± 2.3 years) and 50 older adults (68.1 ± 5.1 year) walked on a treadmill under baseline, three randomized white noise conditions (subthreshold, threshold, and supra-threshold), and a post-condition (Norm_Post_, approximately 15 min after stimulation). Linear mixed-effects models assessed spatiotemporal gait parameters, margin of stability, and gait variability.

**Results:**

Older adults exhibited significantly shorter stride length (ES = 0.28) and greater variability across all gait parameters than younger adults (ES = 0.06–0.22). Auditory white noise significantly affected mean gait parameters, with age-dependent responses observed for most parameters. During subthreshold stimulation, older adults showed significantly reduced cadence (ES = 0.33) and step width (ES = 0.62), and increased stride time (ES = 0.5), support phases (ES = 0.39–0.64), stride length (ES = 0.49), and increased antero-posterior margin of stability (ES = 0.58) compared to baseline. No significant differences were found between the three auditory noise intensities. Several gait parameter changes remained evident in the Norm_Post_ condition with some showing greater changes from baseline than during stimulation.

**Conclusion:**

Auditory white noise stimulation was associated with changes in several gait parameters in older adults that were not observed in younger adults, suggesting age-dependent responses to auditory noise. The persistence of some effects beyond stimulation warrants further investigation into potential underlying sensorimotor and attentional mechanisms. While auditory white noise represents a non-invasive and easily implementable form of sensory stimulation, further studies are needed to establish its effects on gait strategy and fall risk, particularly during overground walking and in clinical populations.

## 1 Introduction

By 2050, the World Health Organisation predicts that over two billion people worldwide will be aged 60 years and above (WHO. World Health Organisation). In Europe, more than 22% of the population is aged 65 years and older, and this number continues to rise (World Bank Group, 2025). Walking is the predominant constituent of human locomotion and is essential for autonomy, wellbeing, and participation in daily life, particularly as we age (Studenski et al., 2011). However, maintaining safe and stable walking becomes increasingly challenging with advancing age, with approximately 28%–35% of older adults falling at least once per year, with falls being a leading cause of injury (WHO, 2007). These incidents frequently result in loss of independence, reduced quality of life, and elevated healthcare costs (WHO, 2007). To address this burgeoning challenge, various prevention strategies, including multifactorial and exercise-based programs, have been developed to enhance gait control and reduce fall risk (Guirguis-Blake et al., 2024). However, such programs are resource- and personnel-intensive, often suffer from poor adherence, and in many cases are impractical and inaccessible for many older individuals (Jung et al., 2023; Sherrington et al., 2017). Therefore, low-cost, practical strategies to promote safe and stable gait at scale are a real and pressing public health priority. Ultimately, promoting stable gait as a vital and sustainable activity requires accessible solutions that support dynamic balance, towards improving overall quality of life in older adults.

Age-related changes in sensory and physical function can contribute to impairments in gait and balance (Iosa et al., 2014; Clark, 2015). These impairments can manifest across distinct aspects of gait, including adaptations in gait strategy, reflecting the ability to maintain stability during locomotion. For example, hearing loss has been associated with slower gait speeds, shorter stride lengths, and increased gait variability (Frosolini et al., 2025), all of which are commonly linked to reduced stability and an elevated risk of falling (McGibbon, 2003). Similarly, reduced visual acuity can compromise the sensory input required for safe navigation and effective balance control (Wieczorek et al., 2024). These examples illustrate how age-associated sensory changes may contribute to gait and balance impairments, although sensory function varies considerably among older adults. Accordingly, there is growing interest in preventive strategies aimed at enhancing sensorimotor function to improve balance and gait strategy in aging populations.

Introducing noise through visual, tactile, or auditory channels has been shown to improve sensorimotor performance, including stepping precision and postural stability in both healthy and clinical populations (Mulavara et al., 2011; Priplata et al., 2002; Pal et al., 2010). Emerging evidence also suggests that older adults may particularly benefit from noise stimulation, as it appears to boost weakened sensory signals and enhance motor outputs (Mulavara et al., 2011; Priplata et al., 2002). Noise can be administered either subthreshold (below conscious perception (Lipsitz et al., 2015; Lugo et al., 2008; Wuehr et al., 2016)), or suprathreshold (noticeable (Simeonov et al., 2011; Aboutorabi et al., 2018)), with some evidence emerging that subthreshold noise stimulation may benefit balance control, while suprathreshold noise may not necessarily provide the same effects (Simeonov et al., 2011; White et al., 2018). Although the exact neurophysiological mechanisms underlying these functional modifications remain unclear, stochastic resonance (SR) has been proposed as one potential explanation. Here, it is thought that SR facilitates the detection and processing of a weak signal that would otherwise be imperceptible due to noise and signal degradation (White et al., 2018; Moss et al., 2004). Specifically, SR describes the phenomenon whereby an optimal level of random noise enhances the detection and transmission of weak signals in nonlinear systems such as the human sensorimotor system (Moss et al., 2004; McDonnell and Abbott, 2009). Interestingly, SR is not thought to be limited to the sensory modality to which the noise is applied. Studies have shown crossmodal effects, where auditory noise, for example, can enhance sensory perception in other modalities (Lugo et al., 2008; Manjarrez et al., 2007). Moreover, the concept of SR overlaps theoretically with movement variability (White et al., 2018), suggesting that a certain level of noise or fluctuation is not inherently negative but may be essential for adaptability and system optimization. Nevertheless, SR is unlikely to be the only mechanism through which auditory noise may influence sensorimotor performance. Alternative explanations include noise-induced changes in attention or arousal (Egeland et al., 2023), as well as enhanced multisensory integration (Lugo et al., 2008), which may alter the processing and use of sensory information for motor control. While the mechanisms underlying these effects remain uncertain, growing evidence across sensory modalities suggests that externally applied noise can influence sensorimotor function.

The addition of tactile noise has been extensively studied (Lipsitz et al., 2015; Simeonov et al., 2011; Aboutorabi et al., 2018; Priplata et al., 2003; Chen et al., 2020), however, the application of auditory noise during gait, especially in older adults, remains relatively unexplored. Previous studies on auditory white noise and balance have predominantly focused on static conditions, demonstrating potential improvements in postural stability (White et al., 2018; Yashima et al., 2021; Kelty-Stephen and Dixon, 2013). However, these findings cannot be directly translated to walking, which involves distinct and continuously changing sensorimotor demands (Reimann et al., 2018). Preliminary evidence suggests that continuous auditory white noise can also influence postural control during walking in older adults (Basta et al., 2023), providing initial support for extending this approach to dynamic conditions. Furthermore, it is unclear whether the impact of auditory noise on gait is specific to the noise intensity, or whether effects persist beyond the period of stimulation. Addressing these questions is important for personalizing stimulation parameters, while also offering these at scale for gait control modifications.

Building on these findings, we investigated the effects of auditory white noise on gait parameters and whether these effects differed according to age and stimulation intensity or persisted following stimulation. We expected subthreshold stimulation to more closely approximate an optimal noise level that facilitates weak sensory signal processing, whereas higher intensities may provide less benefit or interfere with signal processing. We therefore tested three hypotheses: 1) subthreshold auditory white noise exhibits greater enhancement of gait strategy in older compared to younger subjects, 2), subthreshold auditory white noise has a greater impact on gait strategy than threshold and supra-threshold noise, and 3) the effects of auditory white noise continue beyond the period of stimulation.

## 2 Materials and methods

### 2.1 Participants

This intervention study was approved by the ETH Zurich Ethics Commission (EK 2020-N-78) and conducted in accordance with the Declaration of Helsinki. All participants provided written informed consent prior to participation in this study.

No *a-priori* sample size calculation was performed specifically for the present study, as participants were recruited as part of a larger experimental protocol. A total of 100 young and older healthy adults were included in this study (Table 1). Inclusion criteria were a) ability to walk on a treadmill for at least 30 min, b) no acute or chronic neurological or orthopaedic conditions, c) absence of acute pain, d) no use of medications affecting balance, e) no vertigo or balance issues, f) no use of hearing aids, g) no joint replacement within the last 18 months, and h) no alcohol consumption within 24 h prior to testing.

**TABLE 1:** Anthropometrics of young and older participants in mean (SD).

Parameter	Young adults	Older adults
Number of subjects	50	50
Gender	20 M, 30 F	23 M, 27 F
Age [years]	23.8 (2.3)	68.1 (5.1)
Height [cm]	173.0 (10.1)	168.8 (8.3)
Weight [kg]	67.8 (12.3)	69.0 (14.7)
Gait speed [cm/s]	102.7 (12.8)	91.4 (15.3)

### 2.2 Experimental setup

After a short familiarization period on the treadmill, the gait speed was gradually adjusted based on participant feedback until each participant reported a comfortable, self-selected speed that could be maintained throughout the gait trials. After that, the participants first completed a 6 minute baseline gait trial without any auditory stimulation (Norm_Pre_). This was followed by 6 minutes of continuous walking during which white noise was presented at three different volumes: sub-threshold condition (White_35db_), threshold condition (White_44db_), supra-threshold condition (White_65db_), with each volume lasting 2 minutes and delivered in a randomized order. The ambient sound level in the laboratory was assessed during measurement system operation and used to define the experimental stimulation levels. Based on these laboratory-specific conditions, the auditory white noise levels of 35, 44, and 65 dB were selected to represent the subthreshold, threshold, and suprathreshold conditions, respectively. These levels were predefined for the experimental setting and were not individualized according to participants’ auditory perception thresholds. Finally, after approximately 15 min break, participants walked for an additional 6 minutes without noise to complete the post-testing condition (Norm_Post_), to allow testing of possible after-effects (Figure 1).

**FIGURE 1:**
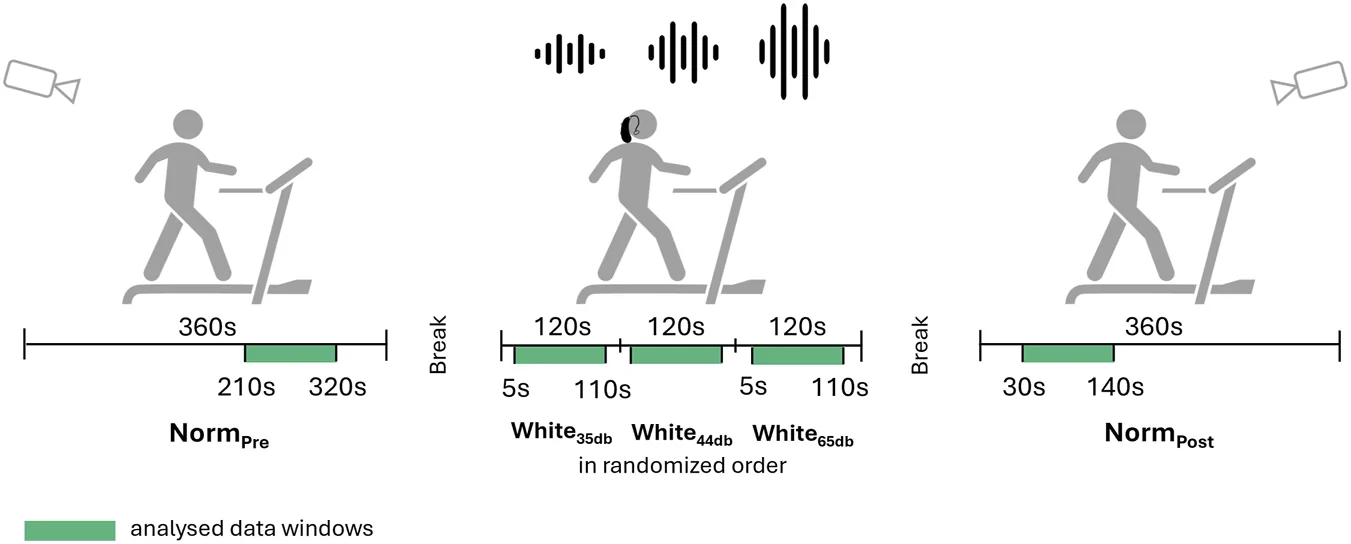
Experimental setup with 6 minutes baseline walking on the treadmill (Norm_Pre_), then 2 minutes of continuous walking under each of three different white noise conditions applied with hearing aids in a randomized order (White_35db_, White_44db_, White_65db_), and Norm_Post_ for post testing condition. Analysed data windows are depicted in green.

### 2.3 Measurement systems

For motion capture, 61 reflective markers were placed on specific anatomical landmarks (Supplementary Material 1. Marker Set), captured using 14 infrared cameras (VICON Motion Systems Ltd., Oxford, United Kingdom) at a sampling rate of 200 Hz. Participants were equipped with bilateral hearing aids (Phonak Audéo MR/RT, Sonova AG, Switzerland), each featuring an integrated audiometer. The devices were wirelessly connected to a computer via Bluetooth to binaurally and continuously deliver white noise at varying intensity levels (description see under 2.2 Experimental setup). Gait trials were conducted on a treadmill (h/p/cosmos quasar, h/p/cosmos sports and medical GmbH, Germany) while wearing a safety harness.

### 2.4 Data processing

To ensure the analysis focused on steady-state walking avoiding biased familiarization, and all the trials had the same length, a Norm_Pre_ window was cut from 210 to 320 s (from the 360 s measurement) to ensure steady-state and regular gait data, whereas Norm_Post_ trials were cut from 30 to 140 s, both yielding 110 s of usable data per trial. For the noise conditions, the initial and final 5 s of each 2-min segment were omitted to avoid transients, also resulting in 110 s of analysed gait data per condition (Figure 1).

Marker trajectory data were processed using a low-pass filter (Butterworth, fourth order, bi-directional, 25 Hz cut-off frequency). Gait events, including heel strikes and toe-offs, were detected using a foot velocity-based algorithm (O'Connor et al., 2007; Kim et al., 2025). Here, foot marker trajectories were used to evaluate the vertical velocity of the foot, which enabled the identification of heel strike and toe-off events. These events were controlled for consistency across strides using a combination of custom Matlab scripts (2021b, Mathworks, USA) as well as visual inspection. Strides with incorrectly identified gait events were excluded from further analysis. A stride was defined as the interval in time and space between two successive heel strikes of the same foot. For each leg and stride, key spatiotemporal gait parameters including stride time, single- and double-limb support time, stride length, and step width were computed. Shorter strides and wider steps were considered indicative of a more cautious gait strategy. Temporal parameters were interpreted in conjunction with spatial changes as adaptations in gait timing and support strategy. The mean and standard deviation (SD) were calculated, with SD values serving as an indicator of gait variability and reflecting the consistency of gait patterns. As both increased and reduced variability may have different functional implications depending on the context and population (Ravi et al., 2020), changes in gait variability were interpreted descriptively rather than as inherently beneficial or detrimental. After performing paired samples t-tests, no statistically significant differences were observed between parameters obtained from left and right limbs. Therefore, subsequent analyses were performed using right-limb data only.

#### 2.4.1 Margin of stability

Gait stability was evaluated using the inverted pendulum model described by Hof, Gazendam (Hof et al., 2005). To estimate the body’s instantaneous centre of mass ( CoM ), the centroid of seven reflective markers placed on the pelvis was used (Supplementary Material 1). The extrapolated centre of mass ( XCoM ) was then computed using the equation:

$$XCoM=CoM+\frac{{CoM}_{v}}{\sqrt{\frac{g}{l}}}$$

where ${CoM}_{v}$ represents the velocity of the CoM adjusted for treadmill walking, g is the gravitational acceleration $g=9.81\;\frac{m}{s^{2}}$ , and l is the leg length, calculated as the vector distance from the CoM to the midpoint between the ankle markers on the malleoli. The margin of stability ( MoS ) at any given moment describes the mechanical stability of a system and is positive when the extrapolated centre of mass ( XCoM ) remains within the base of support ( BoS ).

MoS=BoS−XCoM

MoS was determined at heel strike in both antero-posterior ( ${MoS}_{AP}$ ) and medio-lateral directions ( ${MoS}_{ML}$ ), as this instance influences foot placement for the upcoming stance phase (Watson et al., 2021; Hof, 2008; Sangeux et al., 2024). The BoS boundaries were identified using the first metatarsal marker for the antero-posterior direction and the fifth metatarsal marker for the medio-lateral direction. MoS was interpreted as measure describing the relationship between the XCoM and the boundaries of the BoS . As MoS is influenced by walking strategy and spatiotemporal gait characteristics, changes in MoS were interpreted as reflecting differences in dynamic gait control and walking strategy.

### 2.5 Statistical analysis

Multiple linear mixed-effects models were separately fitted for the mean and standard deviation of each gait parameter as dependent variables: cadence (mean only), stride time, single- and double-limb support time, stride length, step width, MoS_AP_, and MoS_ML_. Model assumptions were assessed by examining residual normality and homoscedasticity and were considered adequately met. Age group (young, older) and noise conditions (Norm_Pre_, White_35db_, White_44db_, White_65db_, Norm_Post_) were included as fixed effects, along with their interaction. Participant ID was included as a random effect to account for inter-individual variability. To test our hypotheses, *post hoc* contrasts were constructed separately for both the young and older groups: 1) White_35db_ = Norm_Pre_, 2) White_35db_ = ½*White_44db_ + ½*White_65db_, and 3) Norm_Post_ = Norm_Pre_. These contrasts characterize within-group responses and were not used as direct tests of differences between age groups; age-dependent differences in response were evaluated using the group × condition interaction. The false discovery rate (FDR) approach (Benjamini and Hochberg, 1995) was used to adjust *p-values* for multiple comparisons and it was applied separately across all tests of mean gait parameters and across all tests of gait parameter standard deviations. Partial eta-squared (ηp^2^) was used to quantify effect sizes for overall fixed effects (group, condition, group × condition) comparison from the linear mixed models. As it is evaluated using F-statistics - ηp^2^ is a variance-partitioning statistic and more suitable for multi-degree-of-freedom factors tests. Partial ηp^2^ values of approximately 0.01, 0.06, and 0.14 were interpreted as small, medium, and large effects, respectively. For pairwise contrasts, Cohen’s d was calculated (d = 2t/√df) as the standardized effect size, consistent with its established use for single-degree-of-freedom mean comparisons and enabling direct comparison with previous literature on gait and balance. Statistical significance was defined as *p ≤ 0.05* and all analyses were conducted using RStudio (v2024.04.2, R Core Team, Austria).

## 3 Results

Older adults exhibited significantly shorter stride length (p < 0.001, ES = 0.28) (Table 2) as well as greater variability (ES = 0.06–0.22), reflected by higher standard deviations across all gait parameters (Supplementary Table S2). The main effect of white noise condition was significantly different for all mean gait parameters with highest ES for MoS_AP_ (p < 0.001, ES = 0.23), step width (p < 0.001, ES = 0.20), and double limb support time (p < 0.001, ES = 0.16, Table 2). For variability parameters, only MoS_AP_ was significantly different (p < 0.001, ES = 0.08, Supplementary Table S2). Interaction effects of age and noise condition for mean gait parameters were significantly different for all gait parameters except step width and MoS_ML_ with highest effect in cadence (p < 0.001, ES = 0.09, Table 2) and for variability gait parameters, only MoS_ML_ was significantly different (p = 0.025, ES = 0.03, Supplementary Table S2). Overall, the largest effect sizes were observed for the main effect of condition across several mean gait parameters, whereas group × condition interaction effects were small to medium. Age-group effects varied across parameters, with particularly large effects observed for stride length and for some measures of gait variability.

**TABLE 2:** Effects of age group, auditory noise condition, and their interaction on mean gait parameters.

Gait parameter	Effect	F (df1, df2)	p-value	ES (ηp^2^)
Cadence [steps/min]	Group	1.53 (1, 98)	0.276	0.02
	Condition	12.51 (4, 392)	**<0.001**	**0.11**
	Group:Condition	9.11 (4, 392)	**<0.001**	**0.09**
Stride time [s]	Group	1.18 (1, 98)	0.337	0.01
	Condition	7.60 (4, 392)	**<0.001**	**0.07**
	Group:Condition	7.65 (4, 392)	**<0.001**	**0.07**
Single support [s]	Group	3.05 (1, 98)	0.112	0.03
	Condition	3.13 (4, 392)	**0.026**	**0.03**
	Group:Condition	7.57 (4, 392)	**<0.001**	**0.07**
Double support [s]	Group	0.30 (1, 98)	0.584	0.00
	Condition	18.86 (4, 392)	**<0.001**	**0.16**
	Group:Condition	3.07 (4, 392)	**0.026**	**0.03**
Stride length [cm]	Group	38.37 (1, 98)	**<0.001**	**0.28**
	Condition	10.07 (4, 392)	**<0.001**	**0.09**
	Group:Condition	6.40 (4, 392)	**<0.001**	**0.06**
Step width [cm]	Group	0.84 (1, 98)	0.405	0.01
	Condition	24.39 (4, 392)	**<0.001**	**0.20**
	Group:Condition	2.23 (4, 392)	0.092	0.02
MoS_AP_ [cm]	Group	0.45 (1, 98)	0.524	0.00
	Condition	30.10 (4, 392)	**<0.001**	**0.23**
	Group:Condition	4.78 (4, 392)	**0.002**	**0.05**
MoS_ML_ [cm]	Group	3.95 (1, 98)	0.074	0.04
	Condition	4.89 (4, 392)	**0.001**	**0.05**
	Group:Condition	1.07 (4, 392)	0.405	0.01

Bold values indicate statistically significant differences (p<0.05).

### 3.1 Effects of subthreshold auditory white noise on gait

In older adults, mean cadence and step width significantly decreased by 1.22 steps/min (p = 0.003, ES = 0.33) and 0.7 cm (p < 0.001, ES = 0.62), respectively in the White_35db_ noise condition compared to Norm_Pre_ (Tables 2–4). In contrast, stride time increased by 0.03 s (p < 0.001, ES = 0.5) and single- and double-limb support time increased by 0.01 s (p < 0.001, ES = 0.39, ES = 0.64), stride length by 1.9 cm (p < 0.001, ES = 0.49), and MoS_AP_ by 0.9 cm (p < 0.001, ES = 0.58) (Tables 2–4) for White_35db_ compared to baseline. Among younger adults, only step width decreased significantly by 0.3 cm (p = 0.016, ES = 0.28), while double-limb support time increased by 0.01s (p = 0.022, ES = 0.26) and MoS_AP_ by 0.4 cm (p = 0.015, ES = 0.28, Tables 2–4) with the introduction of White_35db_ compared to baseline.

**TABLE 3:** Gait parameters (mean (SD)) for the five different conditions in young and older adults.

Parameter	Young				
	Norm_Pre_	White_35db_	White_44db_	White_65db_	Norm_Post_
Cadence [steps/min]	103.97 (8.39)	104.37 (8.28)	104.72 (8.54)	104.46 (8.60)	103.70 (8.37)
Stride time [s]	1.17 (0.11)	1.17 (0.11)	1.17 (0.11)	1.17 (0.11)	1.17 (0.11)
Single support [s]	0.42 (0.03)	0.42 (0.03)	0.42 (0.03)	0.42 (0.03)	0.42 (0.03)
Double support [s]	0.32 (0.07)	0.33 (0.03) *	0.33 (0.03)	0.33 (0.03)	**0.33 (0.06) ††**
Stride length [cm]	121.2 (11.6)	121.7 (12.0)	121.4 (12.2)	121.6 (11.9)	121.6 (12.3)
Step width [cm]	8.7 (2.8)	8.4 (2.9) *	8.4 (2.8)	8.5 (2.8)	**8.2 (3.0) †††**
MoS_AP_ [cm]	17.3 (3.4)	17.7 (3.4) *	17.6 (3.5)	17.6 (3.5)	**18.0 (3.5) †††**
MoS_ML_ [cm]	9.8 (1.6)	9.9 (1.6)	9.8 (1.6)	9.8 (1.7)	**10.0 (1.7) ††**

Parameter	Older adults				
	Norm_Pre_	White_35db_	White_44db_	White_65db_	Norm_Post_
Cadence [steps/min]	108.13 (10.91)	106.90 (11.14) **	106.32 (11.00)	107.04 (11.46)	**104.97 (11.78) †††**
Stride time [s]	1.12 (0.10)	1.15 (0.12) ***	1.15 (0.12)	1.15 (0.12)	**1.16 (0.13) †††**
Single support [s]	0.40 (0.03)	0.41 (0.03) ***	0.41 (0.04)	0.41 (0.04)	**0.42 (0.04) †††**
Double support [s]	0.31 (0.06)	0.32 (0.07) ***	0.32 (0.06)	0.32 (0.06)	**0.32 (0.06) †††**
Stride length [cm]	103.9 (14.5)	105.8 (13.3) ***	106.3 (13.8)	105.6 (13.6)	**106.8 (13.6) †††**
Step width [cm]	9.5 (3.5)	8.8 (3.2) ***	9.1 (3.2)	9.0 (3.2)	**8.5 (3.4) †††**
MoS_AP_ [cm]	16.2 (3.8)	17.1 (3.9) ***	17.4 (3.9)	17.1 (4.0)	**17.9 (4.1) †††**
MoS_ML_ [cm]	10.4 (1.5)	10.4 (1.5)	10.6 (1.5)	10.5 (1.6)	**10.5 (1.6) †**

Post-hoc comparison tests highlighted in bold show significant differences between White_35db_ and Norm_Pre_ (*p < 0.05, **p < 0.01, ***p < 0.001), and between Norm_Post_ and Norm_Pre_ conditions (†p < 0.05, ††p < 0.01, †††p < 0.001). Grey cells denote significant group differences. MoS_AP_: antero-posterior margin of stability; MoS_ML_: medio-lateral margin of stability.

**TABLE 4:** Statistical results of first hypothesis: subthreshold auditory white noise exhibits greater enhancement of gait stability in older compared to young subjects.

Group	Parameter	Estimate	Se	df	t-value	p-value	Cohen’s d
Young	Cadence [steps/min]	0.40	0.37	392	1.09	0.475	0.11
Older	Cadence [steps/min]	−1.23	0.37	392	−3.29	**0.003**	**0.33**
Young	Stride time [s]	0.00	0.00	392	0.64	0.642	0.06
Older	Stride time [s]	0.02	0.00	392	4.92	**<0.001**	**0.50**
Young	Single support time [s]	0.00	0.00	392	−0.16	0.888	0.02
Older	Single support time [s]	0.01	0.00	392	3.85	**<0.001**	**0.39**
Young	Double support time [s]	0.00	0.00	392	2.59	**0.022**	**0.26**
Older	Double support time [s]	0.01	0.00	392	6.38	**<0.001**	**0.64**
Young	Stride length [cm]	0.52	0.39	392	1.32	0.346	0.13
Older	Stride length [cm]	1.91	0.39	392	4.87	**<0.001**	**0.49**
Young	Step width [cm]	−0.30	0.11	392	−2.73	**0.016**	**0.28**
Older	Step width [cm]	−0.68	0.11	392	−6.11	**<0.001**	**0.62**
Young	MoS_AP_ [cm]	0.44	0.16	392	2.77	**0.015**	**0.28**
Older	MoS_AP_ [cm]	0.91	0.16	392	5.75	**<0.001**	**0.58**
Young	MoS_ML_ [cm]	0.11	0.06	392	1.91	0.114	0.19
Older	MoS_ML_ [cm]	0.04	0.06	392	0.68	0.630	0.07

SE: standard error; df: degrees of freedom; MoS_AP_: margin of stability in antero-posterior direction, MoS_ML_: margin of stability in medio-lateral direction.

Bold values indicate statistically significant differences (p<0.05).

No significant changes in gait variability were found between White_35db_ and Norm_Pre_ for young and older adults (Supplementary Tables S1 - S3).

### 3.2 Effects of auditory white noise threshold on gait

No significant differences were observed between White_35db_, White_44db_, and White_65db_ conditions across any gait parameter in both young and older adults (Table 2; Supplementary Tables S4 and S5).

### 3.3 Effects of auditory white noise beyond the period of stimulation

In older adults, effects beyond the period of stimulation in Norm_Post_ compared to Norm_Pre_ showed significant decreases in mean cadence (3.16 steps/min, p < 0.001, ES = 0.86) and step width (1 cm, p < 0.001, ES = 0.87) (Table 2; Figure 2; Supplementary Table S6). Additionally, mean stride time increased by 0.04 s (p < 0.001, ES = 0.73), single-limb support by 0.02 s (p < 0.001, ES = 0.6), double-limb support time by 0.01 s (p < 0.001, ES = 0.77) stride length by 2.9 cm (p < 0.001, ES = 0.76), MoS_AP_ by 1.7 cm (p < 0.001, ES = 1.05), and MoS_ML_ by 0.1 cm (p = 0.022, ES = 0.26, Table 2; Supplementary Table S6). In younger adults, mean step width decreased significantly by 0.5 cm (p < 0.001, ES = 0.51), while double-limb support time increased by 0.01 s (p = 0.003, ES = 0.34), MoS_AP_ by 0.7 cm (p < 0.001, ES = 0.5), and MoS_ML_ by 0.2 cm (p = 0.002, ES = 0.35, Table 2; Supplementary Table S6).

**FIGURE 2:**
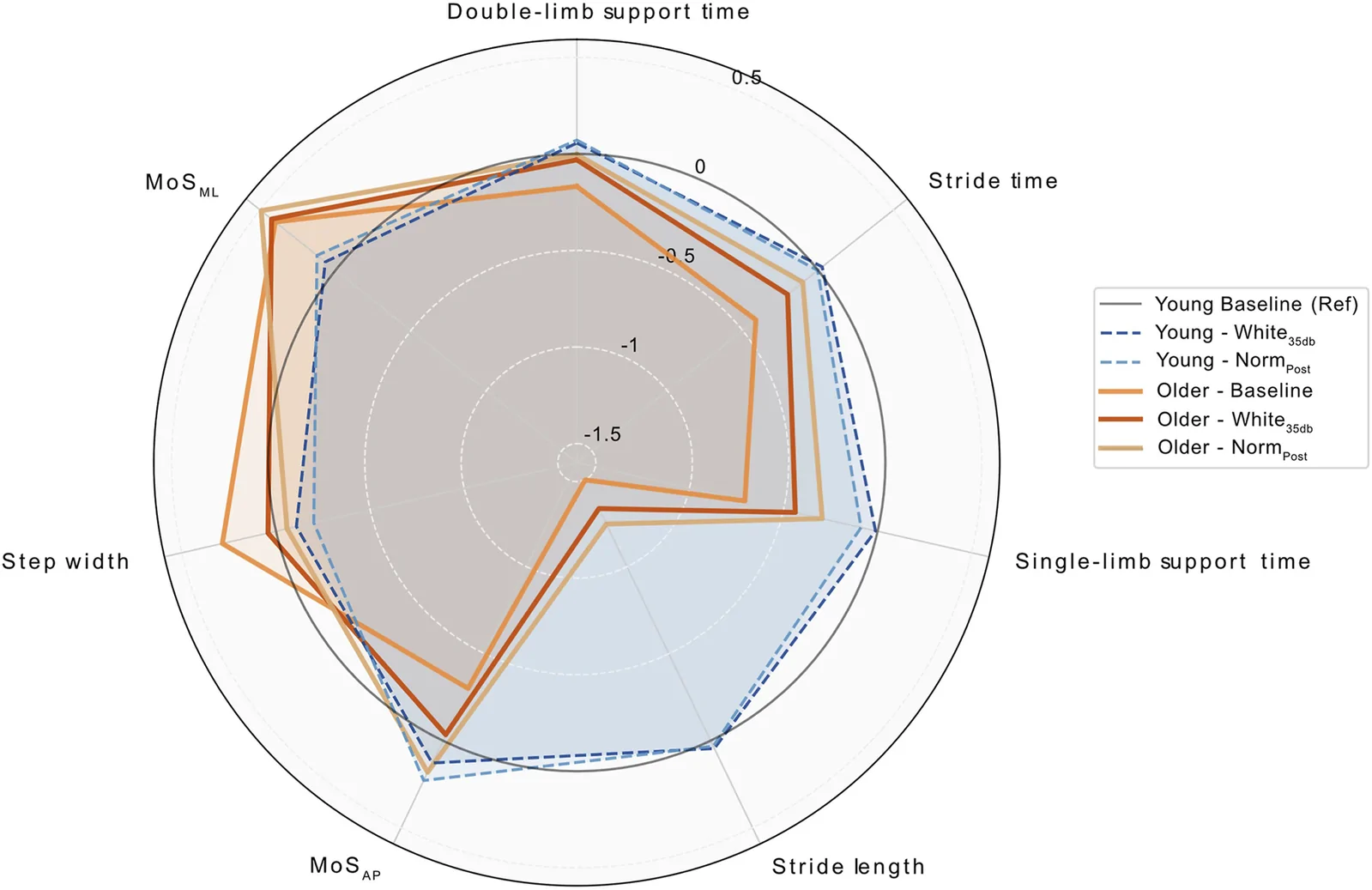
Mean gait parameters for young participants presented as z-scores (contours indicate standard deviations) at baseline compared to noise White_35db_ and post-noise (Norm_Post_), as well as older adults at baseline and under subthreshold auditory white noise stimulation (White_35db_), and Norm_Post_. MoS_AP_: margin of stability in antero-posterior direction, MoS_ML_: margin of stability in medio-lateral direction.

For gait variability, only MoS_AP_ showed a significant reduction, 0.2 cm in young (p = 0.042, ES = 0.31) and 0.2 cm in older adults (p < 0.001, ES = 0.47, Supplementary Tables S1 and S7).

## 4 Discussion

This study investigated the effects of auditory white noise stimulation on gait parameters and gait strategy in young and older adults. In older adults, subthreshold auditory white noise stimulation altered several *mean* gait parameters, such as increased stride time, support phases, and stride length, along with a reduced step width and increased MoS_AP_. In contrast, in younger adults, only double limb support time, step width, and MoS_AP_ were significantly different in White_35db_ condition compared to baseline, indicating a shift in gait strategy. Finally, several *mean* gait adaptations persisted after the auditory white noise stimulation was removed, indicating potential short-term after-effects of the stimulation. Except variability of MoS_AP_, other gait *variability* parameters remained largely unaffected.

### 4.1 Differences between young and older adults

Older adults exhibited significantly greater gait variability compared to younger adults, consistent with previous findings (Schrager et al., 2008; Menz et al., 2003; Hausdorff, 2005). Increased gait variability often reflects impaired motor control, which can compromise accurate foot placement and the dynamic management of body CoM (Maki, 1997). Both factors are strongly associated with an increased risk of falls in older adults (Maki, 1997).

Furthermore, linear-mixed effects models revealed significant group × condition interactions for six of the eight mean gait parameters (cadence, stride time, single- and double-limb support time, stride length and MoS_AP_ (each with adequate statistical power (≥ 0.81), Supplementary Table S8), indicating that the response to auditory white noise differed between younger and older adults across several aspects of gait. No significant interactions were observed for step width or MoS_ML_. Overall, this pattern supports an age-dependent response to auditory white noise across multiple gait parameters, although the magnitude of the interaction effects was generally small to moderate. Older adults showed adaptations across several gait parameters, whereas fewer changes were observed in younger adults. This may indicate that auditory white noise provides additional sensory information that older adults likely use to adapt their gait strategy, while younger adults may have less need for such adaptations due to more preserved sensorimotor function. Age-related differences in motor control strategies, including a shift from ankle-dominated control toward greater involvement of proximal joints, have previously been reported (Begue et al., 2021). Instead of relying primarily on fine corrective movements at the ankle, they engage hip muscles to compensate for instability, resulting in larger, more proximal movements of the trunk and pelvis. Enhanced sensorimotor feedback through auditory white noise stimulation may help older adults adapting their gait strategy, supporting more precise foot placement and reducing reliance on proximal compensatory movements (Gwerder et al., 2025). Future studies incorporating joint kinematics and muscle activity could investigate whether the observed gait adaptations are accompanied by changes in gait strategies.

### 4.2 Effects of subthreshold auditory white noise on gait

Our findings demonstrate that subthreshold auditory white noise stimulation may lead to multiple adaptations in mean gait parameters in older adults. Specifically, cadence and step width decreased, while stride time, single- and double-limb support times, stride length, and MoS_AP_ all increased. Collectively, these changes indicate a shift in gait strategy toward a more controlled and stable gait pattern, with some gait characteristics changing in the direction observed in younger adults (Figure 2) (Bruijn et al., 2013; Hak et al., 2013).

The observed decrease in cadence accompanied by an increase in stride time, in the absence of changes in gait speed because it was fixed throughout the whole measurement, may reflect a change in temporal gait strategy, potentially related to altered sensorimotor control in older adults (Maki, 1997). The reduction in step width, together with prolonged single- and double-limb support phases indicates a change in gait strategy during auditory white noise stimulation. One possible explanation is that a narrower step width reflects reduced reliance on a wider base of support and potentially improved mediolateral balance (Winter 1995; Wilson et al., 2019). Importantly, the increase in MoS_AP_ indicates s change in relationship between XCoM and the base of support at heel strike and may therefore reflect an altered strategy for controlling forward progression during gait. (Hof et al., 2005; Herssens et al., 2020). Together with the observed changes in cadence, stride time, support times, and stride length, the increase in MoS_AP_ may represent part of an overall adaptation in gait strategy during auditory white noise stimulation.

Although statistically significant, the functional relevance of the observed changes should be interpreted cautiously. Changes in individual gait parameters ranged from approximately 1%–7% during auditory white noise stimulation. Previous work has proposed thresholds for meaningful changes in broader physical performance measures, such as approximately 20 m for the 6-min walking distance in older adults (Perera et al., 2006), but comparable thresholds have not been established for several of the parameters assessed here. Nevertheless, the occurrence across multiple gait parameters suggests a measurable modification of gait strategy that warrants further investigation to determine its functional and clinical relevance. For context, changes in some mean gait parameters with auditory white noise stimulation occurred in a similar direction to those previously reported in older adults following exercise interventions (Lord et al., 1996; Fujita et al., 2021). For example, increased stride length has been associated with enhanced gait stability (Lord et al., 1996). However, this comparison should be interpreted cautiously, as auditory white noise and exercise differ substantially in their mechanisms, duration of exposure, and expected physiological adaptations. While exercise interventions typically require repeated training, the present study identified small, immediate changes in gait parameters following auditory white noise stimulation. These findings raise the question of whether auditory white noise could complement established, exercise-based approaches. Future studies should directly investigate the combined effects of auditory white noise and structured exercise and determine whether such an approach provides any additional or longer-term benefits.

Notably, these adaptations across multiple mean gait parameters were not observed in younger adults. One possible explanation is that older adults may have greater capacity to benefit from additional sensory stimulation due to age-related changes in sensorimotor function (Priplata et al., 2003; Costa et al., 2007), or greater reliance on compensatory attentional resources during walking (Behrmann et al., 2004). Young participants with an inherent redundancy of a healthy system may have multiple possible strategies to adapt to different conditions, so that auditory white noise does not have the same beneficial effect as in older adults (Lubetzky et al., 2020). SR provides one potential framework for explaining age-dependent responses, whereby an optimal level of noise may facilitate the processing of weak sensory signals in nonlinear systems (Moss et al., 2004). However, the precise neural mechanisms underlying SR remain unclear and need further investigation.

Gait variability measures remained largely unchanged, suggesting that auditory white noise stimulation might improve average gait performance without necessarily reducing variability, or perhaps longer than 2 minutes of exposure may be required for effects on variability to emerge (König et al., 2014). Additionally, the different responses observed between mean and variability gait parameters could also hypothetically reflect differences in their underlying neural control. Mean parameters such as step length have been associated with activation of specific brain regions, including the prefrontal cortex, sensorimotor area, limbic system, and hippocampus, whereas gait variability has been partly linked to distinct patterns of neural activity (Wilson et al., 2019). However, whether auditory white noise differentially influences these neural processes through SR, attentional mechanisms, arousal, or altered sensory integration cannot be determined from the present data and warrants further investigation using physiological or neuroimaging measures.

### 4.3 Effects of auditory white noise threshold on gait

No significant differences were observed between subthreshold (White_35db_), threshold (White_44db_), and suprathreshold (White_65db_) white noise intensities across any gait parameter in either age group. Previous studies have demonstrated that suprathreshold external sensory information (auditory, visual, or somatosensory) can enhance motor performance and facilitate motor learning (Rochester et al., 2010; Rochester et al., 2007; Semjen et al., 2000; Heremans et al., 2009). Among these modalities, auditory cueing is often preferred by participants (Rochester et al., 2007) and has been reported to evoke the strongest motor response (Weeks et al., 2001).

The findings of the present study suggest that the beneficial effects of auditory white-noise stimulation may not strongly depend on stimulus intensity under the conditions tested. In other words, increasing the noise intensity from subthreshold to suprathreshold levels did not produce additional gait-related effects. Similar results were found for postural control in standing (Lubetzky et al., 2020). The absence of an intensity-dependent relationship raises the possibility that, within the tested range, precise stimulation intensity may be less critical than initially hypothesized. If confirmed, this could have practical advantages by reducing the need for extensive individualization and allowing the use of lower, less intrusive stimulation intensities that are less likely to interfere with daily activities.

### 4.4 Effects of auditory white noise beyond the period of stimulation

Gait adaptations observed during the subthreshold auditory white noise condition (White_35db_) not only persisted after the stimulation but were even higher than in the baseline condition among older adults. Reductions in cadence and step width were larger than during stimulation, while increases in stride time, support phases, stride length, and MoS_AP_ remained significant. Interestingly, younger adults also exhibited significant post-stimulation effects, particularly in step width and MoS_AP_.

These findings raise the possibility that some of the observed post-stimulation changes may reflect short-term adaptations following auditory white noise exposure, potentially involving changes in sensorimotor attention or integration. Similar after-effects have been documented in balance tasks following noise exposure (Gravelle et al., 2002; Collins et al., 2003), as well as carry-over effects from sensory cueing (Rochester et al., 2010; Rochester et al., 2007). Several mechanisms could contribute to such after-effects. Temporary changes in sensorimotor weighting may alter the relative contribution of sensory input to gait control even after the auditory stimulus is removed (Mendez-Balbuena et al., 2012). Alternatively, auditory stimulation may transiently facilitate attentional allocation to sensory information possibly relevant for gait, with effects persisting beyond the period of stimulation (Egeland et al., 2023). Finally, short-term neural plasticity may also contribute to maintaining stimulation induced adaptations within sensorimotor networks (Pellegrino et al., 2022). These mechanisms are not mutually exclusive and remain speculative. The observation that after-effects were stronger than the effects observed during stimulation in older adults raises the question of whether auditory white noise stimulation could contribute to a priming effect on gait control. Future studies should therefore investigate the time course of post-stimulation changes using repeated assessments and control conditions to determine whether such an effect can be reliably reproduced and how long it persists after stimulation.

### 4.5 Clinical relevance

The findings of this study add to emerging evidence that sensory noise, including auditory white noise, may influence gait control in older adults. Auditory white noise has potential practical advantages, as it is non-invasive, inexpensive, and relatively easy to administer. However, the present findings were obtained under controlled treadmill conditions in healthy older adults following a short stimulation period and therefore do not establish the clinical efficacy of auditory white noise or its applicability to individuals with impaired motor function. Furthermore, the mechanisms underlying the observed effects remain unclear, and optimal stimulation parameters, exposure duration, individual responsiveness, and the persistence of effects require further investigation. While the observed after-effects raise the possibility of effects extending beyond the stimulation period, their reproducibility, duration, and clinical relevance remain to be established. Future longitudinal studies in clinical populations and under ecologically valid walking conditions are needed.

### 4.6 Limitations

Several limitations need to be considered. First, hearing acuity was not objectively assessed, and auditory white noise levels were defined based on laboratory conditions rather than individual perceptual thresholds. Second, the relatively short duration represents a limitation, as optimal duration for auditory white noise exposure remains unknown. While longer duration may produce more pronounced effects, however given that the main aim of this study was to identify whether auditory white noise will impact gait performance (and at which thresholds), the choice of duration was considered to manage participant workload. Third, the study did not include long-term follow-up to assess the duration of auditory noise-induced improvements. Additionally, although condition order was randomized, prolonged treadmill walking may have independently influenced gait parameters through adaptation or fatigue. Fatigue was not directly assessed, and the absence of a time-matched control condition prevents these effects from being fully distinguished from those of auditory stimulation. Finally, while the treadmill setup and use of a harness ensured safety and standardization, it guides participants towards a constant velocity and regular rhythm, hence indirectly providing biased guidance to gait patterns. Overground walking is likely to yield different responses and should be thoroughly tested in future studies. Despite those differences, use of treadmill together with harness continues to be a salient part of movement rehabilitation and therapeutic approaches with many studies investigating the impact of treadmill on daily life gait and balance (Rieger et al., 2024).

## 5 Conclusion

In summary, subthreshold auditory white noise was associated with changes in several gait parameters in older adults, including reduced step width and increased stride time, stride length, and MoS_AP_, whereas those changes were not observed in younger adults. These age-dependent responses suggest that the effects of auditory white noise on gait may differ with advancing age and warrant further investigation into whether age-related changes in sensorimotor function contribute to individual responsiveness. The observed changes may be related to alterations in sensorimotor integration or attentional processes. While auditory white noise represents a non-invasive and easily implementable form of sensory stimulation, its potential to improve gait and reduce fall risk remains to be established. Future studies should investigate the underlying mechanisms, long-term effects, and clinical relevance of auditory white noise during overground walking and in populations with gait or balance impairments.

## Data Availability

The original contributions presented in the study are included in the article/Supplementary Material, further inquiries can be directed to the corresponding author.
