# Supplementary material for: Walking with noise: the impact of auditory white noise stimulation on gait dynamics in young and older adults
Source: Front Bioeng Biotechnol. 2026 Oct 7;14:1910987. doi: 10.3389/fbioe.2026.1910987 (PMC13683698; doi:10.3389/fbioe.2026.1910987)
Supplement: Supplementary file 1 [file DataSheet1.pdf]

frontiers

Supplementary Material

1 Marker set

Name	Segment	Location
RTO1	Foot - right	Caput metatarsale I
RTO3	Foot - right	Metatarsale III
RTO5	Foot - right	Caput metatarsale VI
RFOX	Foot - right	Lateral, not specified (Extramarker)
RHEE	Foot - right	Calcaneus
RMMA	Shank - right	Medial malleolus
RLMA	Shank - right	Lateral malleolus
RTMT	Shank - right	unspecified Tibia, ventral
RTLF	Shank - right	unspecified Fibula, lateral
RTTT	Shank - right	Tuberositas tibea
RTIB	Shank - right	Caput fibulae
RMCO	Thigh - right	Medial femur epicondylus
RLCO	Thigh - right	Lateral femur epicondylus
RTFR	Thigh - right	unspecified Femur, front distal
RTLL	Thigh - right	unspecified Femur, lateral distal
RTLH	Thigh - right	unspecified Femur, lateral proximal
LTO1	Foot - left	Caput metatarsale I
LTO3	Foot - left	Metatarsale III
LTO5	Foot - left	Caput metatarsale VI
LHEE	Foot - left	Calcaneus
LMMA	Shank - left	Medial malleolus
LLMA	Shank - left	Lateral malleolus
LTMT	Shank - left	unspecified Tibia, ventral
LTLF	Shank - left	unspecified Fibula, lateral
LTTT	Shank - left	Tuberositas tibea
LTIB	Shank - left	Caput fibulae
LMCO	Thigh - left	Medial femur epicondylus
LLCO	Thigh - left	Lateral femur epicondylus
LTFR	Thigh - left	unspecified Femur, front distal
LTLL	Thigh - left	unspecified Femur, lateral distal
LTLH	Thigh - left	unspecified Femur, lateral proximal
RASI	Pelvis	Spina illiaca ant. sup. – right

RTMS	Pelvis	Iliaca – right
RPSI	Pelvis	Spina illiaca post. Sup – right
SACR	Pelvis	Sacrum
LPSI	Pelvis	Spina illiaca ant. sup. – left
LTMS	Pelvis	Iliaca - left
LASI	Pelvis	Spina illiaca post. Sup - left
RSHO	Shoulder	Acromion - right
MSTC	Shoulder	Manubrium sterni cranial
CVC7	Shoulder	C7
LSHO	Shoulder	Acromion - left
RWRA	Forearm - right	Proc. styloideus radii
RWUL	Forearm - right	Proc. styloideus ulnae
RFRA	Forearm - right	unspecified forearm, radial
RFUL	Forearm - right	unspecified forearm, ulnae
RMEC	Humerus - right	Medial epicondylus
RLEC	Humerus - right	Lateral epicondylus
RHVT	Humerus - right	unspecified upperarm, ventral
RHLT	Humerus - right	unspecified upperarm, lateral
LWRA	Forearm - left	Proc. styloideus radii
LWUL	Forearm - left	Proc. styloideus ulnae
LFRA	Forearm - left	unspecified forearm, radial
LFUL	Forearm - left	unspecified forearm, ulnae
LMEC	Humerus - left	Medial epicondylus
LLEC	Humerus - left	Lateral epicondylus
LHVT	Humerus – left	unspecified upperarm, ventral
LHLT	Humerus - left	unspecified upperarm, lateral
RFHD	Head	Headband
RBHD	Head	Headband
LFHD	Head	Headband
LBHD	Head	Headband

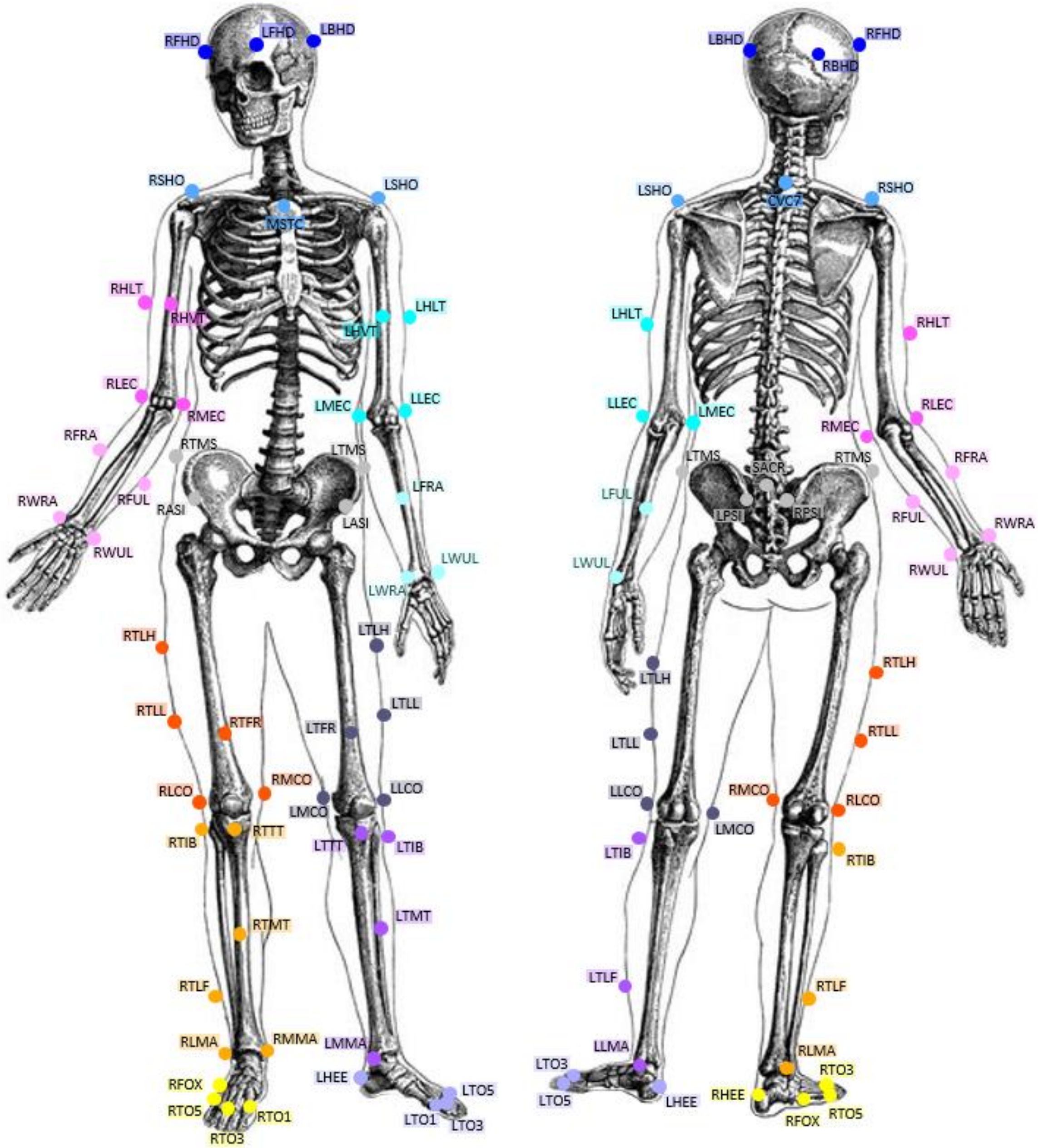
RFHD
LFHD
LBHD
RSHO
LSHO
MSTC
RHLT
RHVT
LHVT
LHLT
RLEC
RMEC
LMEC
LLEC
RFRA
RTMS
LTMS
LFRA
RWRA
RFUL
RASI
LASI
RWUL
LWUL
LWRA
LTLH
RTLH
LTLL
RTLL
RTFR
LTFR
RMCO
LLCO
RLCO
LMCO
RTIB
RTTT
LTTT
LTIB
LTMT
RTMT
RTLF
RLMA
RMMA
LMMA
LHEE
LTO5
RFOX
RTO5
RTO1
LTO1
LTO3
RTO3
LBHD
RFHD
RBHD
LSHO
CVC7
RSHO
LHLT
RHLT
LLEC
LMEC
RMEC
RLEC
LTMS
SACR
RTMS
RFRA
LFUL
LPSI
RPSI
RFUL
RWRA
LWUL
RWUL
LTLH
RTLH
RTLL
LTLL
LLCO
RMCO
RLCO
LMCO
LTIB
RTIB
LTLF
RTLF
LLMA
RLMA
LTO3
RTO3
LTO5
LHEE
RHEE
RTO5
RFOX

2 Statistical analysis

Table S1: Variability parameters (mean (SD)) for the five different conditions in young and older adults. Post-hoc comparison tests analyzed with contrasts highlight significant differences between $Norm_{Post}$ and $Norm_{Pre}$ († $p<0.05$, ††† $p<0.001$). MoS_{AP}: antero-posterior margin of stability; MoS_{ML}: medio-lateral margin of stability.

	**Young**				
	**$Norm_{Pre}$**	**$White_{35db}$**	**$White_{44db}$**	**$White_{65db}$**	**$Norm_{Post}$**
**Stride Time SD [ms]**	15.1 (5.8)	15.6 (8.3)	15.3 (7.3)	15.7 (6.6)	15.3 (6.7)
**Single Support SD [ms]**	8.7 (3.8)	9.0 (4.7)	8.5 (4.2)	8.8 (3.9)	8.4 (3.7)
**Double Support SD [ms]**	11.1 (4.2)	10.6 (3.5)	10.8 (5.0)	11.1 (4.5)	10.6 (3.4)
**Stride Length SD [cm]**	2.8 (0.7)	2.9 (0.9)	2.8 (0.8)	2.8 (0.7)	2.8 (0.7)
**Step Width SD [cm]**	2.0 (0.7)	2.2 (0.8)	2.1 (0.7)	2.2 (2.2)	2.3 (0.7)
**MoS_{AP} SD [cm]**	1.3 (0.4)	1.3 (0.5)	1.2 (0.3)	1.2 (0.3)	**1.1 (0.2)** †
**MoS_{ML} SD [cm]**	0.8 (0.2)	0.8 (0.2)	0.8 (0.2)	0.8 (0.2)	0.8 (0.2)
	**Older adults**				
	**$Norm_{Pre}$**	**$White_{35db}$**	**$White_{44db}$**	**$White_{65db}$**	**$Norm_{Post}$**
**Stride Time SD [ms]**	21.1 (13.6)	20.4 (9.3)	21.0 (10.6)	20.1 (8.8)	21.8 (10.6)
**Single Support SD [ms]**	12.2 (5.4)	12.3 (5.8)	12.5 (6.4)	12.0 (5.9)	12.3 (6.0)
**Double Support SD [ms]**	14.2 (6.3)	13.7 (5.8)	13.9 (5.9)	13.5 (5.6)	13.7 (5.9)
**Stride Length SD [cm]**	3.4 (1.2)	3.3 (0.9)	3.3 (1.0)	3.2 (1.0)	3.3 (1.0)
**Step Width SD [cm]**	2.6 (0.9)	2.4 (0.9)	2.5 (1.0)	2.3 (0.8)	2.6 (1.1)
**MoS_{AP} SD [cm]**	1.8 (0.7)	1.7 (0.7)	1.7 (0.6)	1.6 (0.7)	**1.6 (0.5)** †††
**MoS_{ML} SD [cm]**	1.0 (0.2)	0.9 (0.3)	0.9 (0.2)	0.9 (0.2)	0.9 (0.2)

Table S2: Effects of age group, auditory noise condition, and their interaction on variability gait parameters.

Gait Parameter	Effect	F (df1, df2)	p-value	ES (ηp^2)
**Stride Time SD [s]**	Group	11.09 (1, 98)	**0.005**	**0.10**
	Condition	0.33 (4, 392)	0.860	0.00
	Group:Condition	1.09 (4, 392)	0.510	0.01
**Single Support SD [s]**	Group	13.95 (1, 98)	**0.002**	**0.12**
	Condition	0.34 (4, 392)	0.860	0.00
	Group:Condition	0.92 (4, 392)	0.557	0.01
**Double Support SD [s]**	Group	8.97 (1, 98)	**0.012**	**0.08**
	Condition	1.54 (4, 392)	0.309	0.02
	Group:Condition	1.08 (4, 392)	0.510	0.01
**Stride Length SD [cm]**	Group	8.77 (1, 98)	**0.012**	**0.08**
	Condition	0.98 (4, 392)	0.552	0.01
	Group:Condition	0.84 (4, 392)	0.577	0.01
**Step Width SD [cm]**	Group	6.51 (1, 98)	**0.029**	**0.06**
	Condition	1.67 (4, 392)	0.300	0.02
	Group:Condition	2.24 (4, 392)	0.134	0.02
**MoS_{AP} SD [cm]**	Group	27.11 (1, 98)	**<0.001**	**0.22**
	Condition	8.35 (4, 392)	**<0.001**	**0.08**
	Group:Condition	0.81 (4, 392)	0.577	0.01
**MoS_{ML} SD [cm]**	Group	14.06 (1, 98)	**0.002**	**0.13**
	Condition	1.57 (4, 392)	0.309	0.02
	Group:Condition	3.40 (4, 392)	**0.025**	**0.03**

Table S3: Gait variability results of first hypothesis: $White_{35db}$ = $Norm_{Pre}$. SE: standard error; df: degrees of freedom; MoS_{AP}: margin of stability in antero-posterior direction, MoS_{ML}: margin of stability in medio-lateral direction.

**Group**	**Parameter**	**Estimate**	**SE**	**df**	**t-value**	**p-value**	**Cohen's d**
Young	Stride time SD [s]	0.00	0.00	392	0.63	0.827	0.06
Older	Stride time SD [s]	0.00	0.00	392	-0.82	0.813	0.08
Young	Single support time SD [s]	0.00	0.00	392	0.74	0.813	0.07
Older	Single support time SD [s]	0.00	0.00	392	0.28	0.929	0.03
Young	Double support time SD [s]	0.00	0.00	392	-1.63	0.599	0.16
Older	Double support time SD [s]	0.00	0.00	392	-1.58	0.599	0.16
Young	Stride length SD [cm]	0.02	0.10	392	0.22	0.929	0.02
Older	Stride length SD [cm]	-0.12	0.10	392	-1.27	0.663	0.13
Young	Step width SD [cm]	0.13	0.12	392	1.05	0.723	0.11
Older	Step width SD [cm]	-0.17	0.12	392	-1.40	0.614	0.14
Young	MoS_{AP} SD [cm]	-0.02	0.05	392	-0.40	0.929	0.04
Older	MoS_{AP} SD [cm]	-0.10	0.05	392	-1.81	0.493	0.18
Young	MoS_{ML} SD [cm]	-0.01	0.02	392	-0.68	0.813	0.07
Older	MoS_{ML} SD [cm]	-0.04	0.02	392	-2.04	0.352	0.21

Table S4: Mean gait results of second hypothesis: $White_{35db} = ½*White_{44db} + ½*White_{65db}$. SE: standard error; df: degrees of freedom; MoS_{AP}: margin of stability in antero-posterior direction, MoS_{ML}: margin of stability in medio-lateral direction.

Group	Parameter	Estimate	SE	df	t-value	p-value	Cohen's d
Young	Cadence [steps/min]	0.22	0.32	392	0.68	0.630	0.07
Older	Cadence [steps/min]	-0.22	0.32	392	-0.68	0.630	0.07
Young	Stride time [s]	0.00	0.00	392	-0.58	0.660	0.06
Older	Stride time [s]	0.00	0.00	392	0.46	0.725	0.05
Young	Single support time [s]	0.00	0.00	392	-1.05	0.486	0.11
Older	Single support time [s]	0.00	0.00	392	0.61	0.648	0.06
Young	Double support time [s]	0.00	0.00	392	0.40	0.754	0.04
Older	Double support time [s]	0.00	0.00	392	-0.28	0.814	0.03
Young	Stride length [cm]	-0.24	0.34	392	-0.72	0.630	0.07
Older	Stride length [cm]	0.18	0.34	392	0.54	0.674	0.05
Young	Step width [cm]	0.03	0.10	392	0.32	0.803	0.03
Older	Step width [cm]	0.20	0.10	392	2.07	0.082	0.21
Young	MoS_{AP} [cm]	-0.09	0.14	392	-0.69	0.630	0.07
Older	MoS_{AP} [cm]	0.10	0.14	392	0.75	0.630	0.08
Young	MoS_{ML} [cm]	-0.04	0.05	392	-0.77	0.630	0.08
Older	MoS_{ML} [cm]	0.07	0.05	392	1.49	0.262	0.15

Table S5: Gait variability results of second hypothesis: $White_{35db} = ½*White_{44db} + ½*White_{65db}$. SE: standard error; df: degrees of freedom; MoS_{AP}: margin of stability in antero-posterior direction, MoS_{ML}: margin of stability in medio-lateral direction.

Group	Parameter	Estimate	SE	df	t-value	p-value	Cohen's d
Young	Stride time SD [s]	0.00	0.00	392	-0.10	0.946	0.01
Older	Stride time SD [s]	0.00	0.00	392	0.14	0.929	0.01
Young	Single support time SD [s]	0.00	0.00	392	-1.05	0.723	0.11
Older	Single support time SD [s]	0.00	0.00	392	-0.27	0.929	0.03
Young	Double support time SD [s]	0.00	0.00	392	1.44	0.614	0.15
Older	Double support time SD [s]	0.00	0.00	392	-0.03	0.973	0.00
Young	Stride length SD [cm]	-0.06	0.08	392	-0.67	0.813	0.07
Older	Stride length SD [cm]	-0.03	0.08	392	-0.41	0.929	0.04
Young	Step width SD [cm]	0.02	0.10	392	0.16	0.929	0.02
Older	Step width SD [cm]	-0.02	0.10	392	-0.17	0.929	0.02
Young	MoS_{AP} SD [cm]	-0.04	0.05	392	-0.87	0.806	0.09
Older	MoS_{AP} SD [cm]	-0.02	0.05	392	-0.46	0.929	0.05
Young	MoS_{ML} SD [cm]	0.02	0.02	392	1.07	0.723	0.11
Older	MoS_{ML} SD [cm]	-0.02	0.02	392	-1.19	0.707	0.12

Table S6: Mean gait results of third hypothesis: $Norm_{Post}$ = $Norm_{Pre}$. SE: standard error; df: degrees of freedom; MoS_{AP}: margin of stability in antero-posterior direction, MoS_{ML}: margin of stability in medio-lateral direction.

Group	Parameter	Estimate	SE	df	t-value	p-value	Cohen's d
Young	Cadence [steps/min]	-0.27	0.37	392	-0.73	0.630	0.07
Older	Cadence [steps/min]	-3.15	0.37	392	-8.48	**<0.001**	**0.86**
Young	Stride time [s]	0.00	0.00	392	-0.06	0.954	0.01
Older	Stride time [s]	0.03	0.00	392	7.27	**<0.001**	**0.73**
Young	Single support time [s]	0.00	0.00	392	-1.27	0.367	0.13
Older	Single support time [s]	0.01	0.00	392	5.90	**<0.001**	**0.60**
Young	Double support time [s]	0.00	0.00	392	3.32	**0.003**	**0.34**
Older	Double support time [s]	0.01	0.00	392	7.66	**<0.001**	**0.77**
Young	Stride length [cm]	0.37	0.39	392	0.94	0.556	0.10
Older	Stride length [cm]	2.94	0.39	392	7.49	**<0.001**	**0.76**
Young	Step width [cm]	-0.56	0.11	392	-5.06	**<0.001**	**0.51**
Older	Step width [cm]	-0.96	0.11	392	-8.66	**<0.001**	**0.87**
Young	MoS_{AP} [cm]	0.78	0.16	392	4.95	**<0.001**	**0.50**
Older	MoS_{AP} [cm]	1.64	0.16	392	10.42	**<0.001**	**1.05**
Young	MoS_{ML} [cm]	0.20	0.06	392	3.44	**0.002**	**0.35**
Older	MoS_{ML} [cm]	0.15	0.06	392	2.61	**0.022**	**0.26**

Table S7: Gait variability results of third hypothesis: $Norm_{Post} = Norm_{Pre}$. SE: standard error; df: degrees of freedom; MoS_{AP}: margin of stability in antero-posterior direction, MoS_{ML}: margin of stability in medio-lateral direction.

Group	Parameter	Estimate	SE	df	t-value	p-value	Cohen's d
Young	Stride time SD [s]	0.00	0.00	392	0.26	0.929	0.03
Older	Stride time SD [s]	0.00	0.00	392	0.80	0.813	0.08
Young	Single support time SD [s]	0.00	0.00	392	-0.72	0.813	0.07
Older	Single support time SD [s]	0.00	0.00	392	0.26	0.929	0.03
Young	Double support time SD [s]	0.00	0.00	392	-1.47	0.614	0.15
Older	Double support time SD [s]	0.00	0.00	392	-1.32	0.654	0.13
Young	Stride length SD [cm]	-0.09	0.10	392	-0.90	0.806	0.09
Older	Stride length SD [cm]	-0.06	0.10	392	-0.58	0.844	0.06
Young	Step width SD [cm]	0.25	0.12	392	2.10	0.352	0.21
Older	Step width SD [cm]	0.03	0.12	392	0.26	0.929	0.03
Young	MoS_{AP} SD [cm]	-0.16	0.05	392	-3.11	**0.042**	**0.31**
Older	MoS_{AP} SD [cm]	-0.25	0.05	392	-4.69	**<0.001**	**0.47**
Young	MoS_{ML} SD [cm]	0.02	0.02	392	0.98	0.760	0.10
Older	MoS_{ML} SD [cm]	-0.05	0.02	392	-2.61	0.131	0.26

Table S8: Power calculations for the first hypothesis $White_{35db} = Norm_{Pre}$ for group x condition results.

Parameter	F (4, 392)	p (raw)	p (adj)	ηp²	Power	H1 supported?
**Cadence**	9.11	<0.001	<0.001	0.093	1.00	✓ Yes
**Stride time**	7.65	<0.001	<0.001	0.078	1.00	✓ Yes
**Single support**	7.57	<0.001	<0.001	0.077	1.00	✓ Yes
**Double support**	3.07	0.017	0.03	0.031	0.81	✓ Yes
**Stride length**	6.40	<0.001	<0.001	0.065	0.99	✓ Yes
**MoS_{AP}**	4.78	<0.001	<0.001	0.049	0.96	✓ Yes
**Step Width**	2.23	0.065	0.09	0.023	0.66	✗ No
**MoS_{ML}**	1.07	0.371	0.40	0.011	0.34	✗ No
